# A novel framework for studying human aging: trophoblast stem cell–syncytiotrophoblast platform as a physiologically relevant and accelerated model of cellular aging

**DOI:** 10.1093/lifemedi/lnag027

**Published:** 2026-07-11

**Authors:** Qingqing Zhang, Elison Po Wa Lee, Baicheng Lin, Timothy Theodore Ka Ki Tam, Zhen Feng, Pentao Liu

**Affiliations:** School of Biomedical Sciences, Li Ka Shing Faculty of Medicine, The University of Hong Kong, Hong Kong 999077, China; InnoHK Centre for Translational Stem Cell Biology, Hong Kong Science & Technology Park, Hong Kong Special Administrative Region 999077, China; School of Biomedical Sciences, Li Ka Shing Faculty of Medicine, The University of Hong Kong, Hong Kong 999077, China; InnoHK Centre for Translational Stem Cell Biology, Hong Kong Science & Technology Park, Hong Kong Special Administrative Region 999077, China; InnoHK Centre for Translational Stem Cell Biology, Hong Kong Science & Technology Park, Hong Kong Special Administrative Region 999077, China; InnoHK Centre for Translational Stem Cell Biology, Hong Kong Science & Technology Park, Hong Kong Special Administrative Region 999077, China; School of Biomedical Sciences, Li Ka Shing Faculty of Medicine, The University of Hong Kong, Hong Kong 999077, China; InnoHK Centre for Translational Stem Cell Biology, Hong Kong Science & Technology Park, Hong Kong Special Administrative Region 999077, China

While animal models and *in vitro* cellular systems have been extensively employed to investigate aging, leading to the identification of evolutionarily conserved aging pathways and hallmarks, their direct translational relevance to human physiological aging warrants further elucidation. Current human cellular aging models often rely on extended passaging, exposure to exogenous stressors, or genetic alterations to induce cellular senescence, approaches that may not fully recapitulate the complex cellular dynamics underlying natural aging. To address this longstanding challenge, we recently developed a system in which human trophoblast stem cells (hTSCs) differentiate into syncytiotrophoblasts (STBs), faithfully recapitulating key aspects of *in vivo* trophoblast development in the placenta. Remarkably, this process rapidly manifests multiple established hallmarks of cellular aging within a short time frame [1]. This novel platform offers a physiologically relevant and accelerated model for studying human aging, providing a valuable tool to investigate aging mechanisms and test potential interventions.

The placenta undergoes dynamic cellular changes during development that resemble features commonly associated with aging. A growing body of evidence supports the presence of these features, not only under normal physiological conditions but also in pathological states where these processes are accelerated, often leading to adverse pregnancy outcomes. At the cellular level, these aging features of the placenta are predominantly linked to cellular senescence [2]. Intriguingly, emerging studies suggest that cellular senescence is not merely a passive consequence of aging but may serve as a critical physiological driver of development. For instance, the fusion of cytotrophoblasts (CTBs) to form STBs induces senescence while enabling the continuous repair of the microvillous syncytium throughout pregnancy [2]. Disruption of this tightly regulated process can lead to placental pathologies [2], further supporting the notion that dysregulated aging mechanisms can contribute to pregnancy complications. Moreover, GDF15, a senescence-associated factor produced by trophoblasts, is critically required for the proper development and function of extravillous trophoblasts [3]. These findings underscore the aging-like mechanisms in the placenta and emphasize the need to adopt a multifaceted approach when investigating the aging-related phenomena in trophoblasts.

Recent studies further strengthen the link between placental biology and cellular aging, particularly through the ectopic reactivation of placenta-specific genes in nonplacental tissues. A recent study systematically profiled the dynamic alterations in the epigenomic landscape during human mesenchymal progenitor cell (hMPC) aging. It was reported that alterations in genomic organization, including increased chromatin entropy, epigenetic instability, heterochromatin erosion, euchromatin weakening, and topological compartment switching, led to the aberrant activation of placenta-specific genes and pathways [4]. This observation has led to the hypothesis that pregnancy-specific glycoprotein (PSG) reactivation may not only serve as a biomarker but also actively contribute to cellular aging by exacerbating senescence phenotypes [4]. These findings suggest that age-related epigenetic dysregulation can drive inappropriate expression of placenta-specific genes, offering molecular evidence that placental genetic programs may be co-opted during pathological aging in other tissues.

Further supporting this concept, a complementary study investigated the consequences of sirtuin deficiencies in hMPCs [5]. The authors demonstrated that depletion of any sirtuin family member (*SIRT1*–*SIRT7*) accelerated cellular senescence through widespread disruptions in chromatin architecture. Epigenomic analyses revealed that sirtuin loss triggered rewiring of enhancer–promoter interactions within sirtuin deficiency-sensitive genomic regions (SDSRs), which are enriched in active enhancers and promote *de novo* enhancer–promoter loop formation. Across all sirtuin-deficient models, altered chromatin domain interactions resulted in ectopic activation of *PAPPA* (pregnancy-associated plasma protein A), a placenta-specific gene that promotes senescence phenotypes and serves as a known biomarker of aging [5]. Collectively, these findings highlight how chromatin remodeling during aging can reactivate placenta-specific genes, revealing a potential mechanistic link between physiological placental development and systemic aging processes.

Proteomic profiling of hTSC-to-STB development [1] and of aging plasma [6] revealed overlapping differentially expressed proteins, particularly the upregulation of SERPINE2. Strikingly, other placenta-specific proteins, such as PAPPA [5] and GDF15 [7], are also present at elevated levels in the serum of elderly individuals, providing direct clinical evidence for the reactivation of placental genetic programs along organismal aging. Besides SERPINE2, PAPPA, and GDF15, proteomic studies of aged plasma have identified other trophoblast-related or ‑derived proteins, including IGFBP2, IGFBP4, TIMP1, and INHBA (also known as ACTIVIN A), further underscoring the mechanistic link between placental maturation and organismal systemic aging.

*In vivo*, our single-cell transcriptomic analyses revealed that STBs in human placenta exhibit progressively increasing senescence scores across gestation. This trajectory is characterized by a gradual downregulation of genes involved in cell-cycle and DNA repair, coupled with activation of inflammatory and metabolic pathways typical of aged cells. Importantly, the inferred aging trajectory of STBs closely resembles that observed in aged human tissue stem cells, such as muscle and hematopoietic stem cells, highlighting the physiological relevance of this model [1].

**Figure 1.**
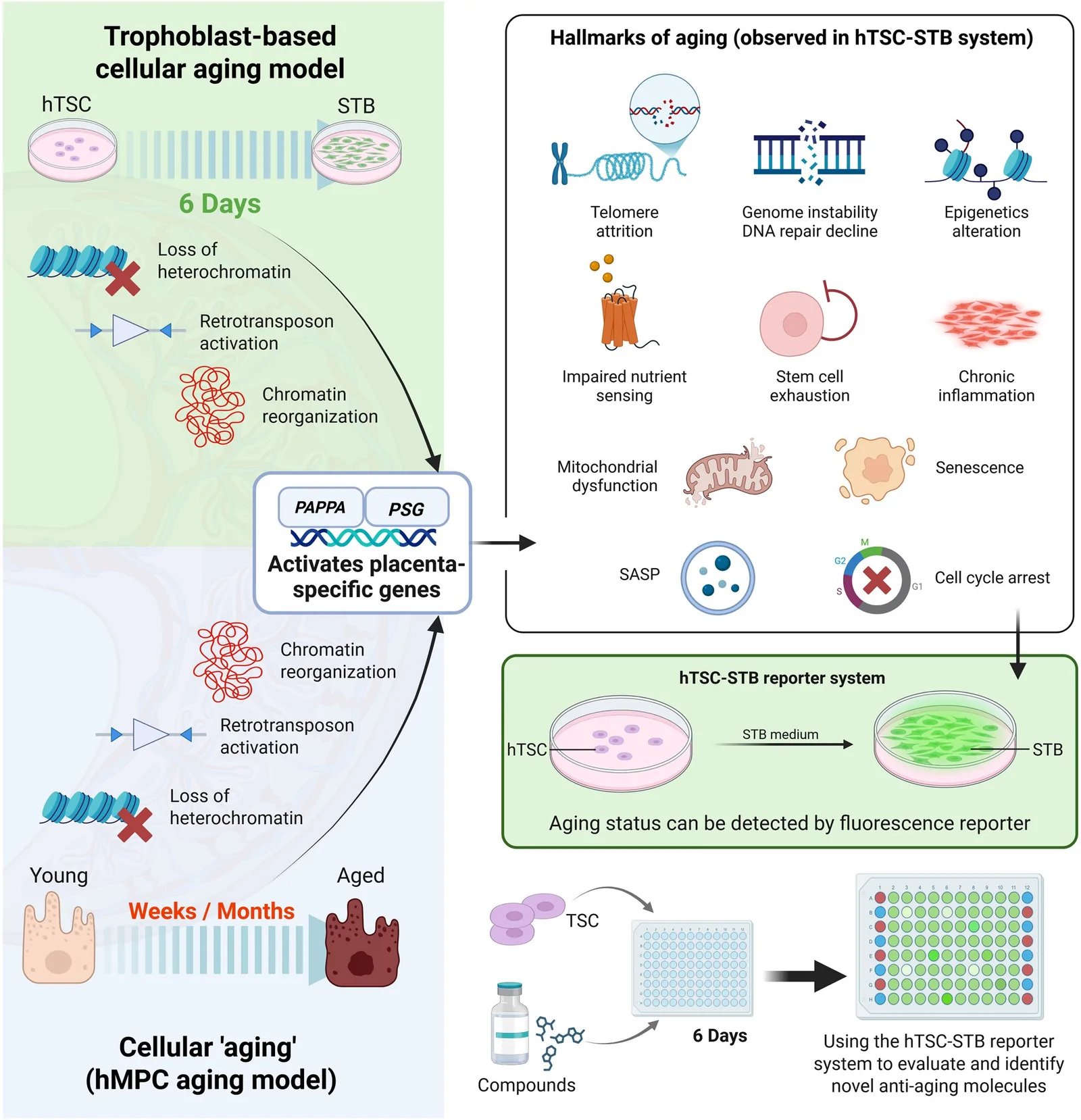
Schematic diagram illustrating the hTSC–STB system as a physiologically relevant and experimentally tractable aging model. Developmental senescence in the syncytiotrophoblast (STB) activates placenta-specific genes, which are also reported in other aging cell types. The system rapidly exhibits key aging hallmarks, making it a valuable tool for aging research. We have further developed an hTSC–STB reporter system, highlighting its potential for the evaluation of known anti-aging molecules and high-throughput screening of anti-aging compounds and for the discovery of novel therapeutic strategies. This figure was made using BioRender.

*In vitro*, hTSCs develop into mature STBs within 6 days, recapitulating key aspects of placental maturation during pregnancy and all cellular aging hallmarks examined. This accelerated development timeline mirrors features of naturally aging tissues and enables the study of aging processes without the need for prolonged culture or artificial stress induction. During hTSC-to-STB development, cells undergo irreversible cell cycle arrest, accompanied by reduced DNA damage responses, increased genomic instability, and extensive epigenetic remodeling, particularly the loss of heterochromatin markers (e.g., H3K9me3 and H3K27me3) and decreased expression of lamins, structural proteins critical for nuclear integrity [1]. Another hallmark of aging, derepression of endogenous retroviruses, is also observed, particularly human endogenous retrovirus K (HERV-K) [1], whose upregulation has been linked to senescence in skin and serum samples [8], further reinforcing the similarity between the hTSC-to-STB model and molecular features of human aging. Notably, during hTSC-to-STB development, STBs spontaneously develop senescence-associated secretory phenotypes (SASPs), marked by secretion of pro-inflammatory cytokines, mitochondrial dysfunction, and metabolic reprogramming, all canonical features of cellular aging [1] (Fig. 1).

A major strength of the hTSC–STB system lies in its experimental tractability, enabling precise manipulation and real-time analysis of aging-related processes. Building upon insights from sirtuin studies, targeted modulation of chromatin regulators may similarly prevent the aberrant derepression of *PAPPA*, *PSGs*, and other aging-associated genes. This approach allows for the mechanistic dissection of how epigenetic dysregulation, chromatin topological remodeling, and senescence progression are functionally interconnected. Moreover, the hTSC–STB platform allows for the rapid evaluation of anti-aging interventions. Treatment with established geroprotective compounds, including the mTOR inhibitors such as rapamycin and INK128, as well as the senolytic agent fisetin, has been shown to reduce senescence markers and delay STB maturation, demonstrating the system’s sensitivity to pharmacological modulation [1]. These findings underscore the model’s utility not only for mechanistic studies but also for high-throughput screening of novel therapeutic candidates aimed at modulating aging pathways.

The hTSC–STB model addresses several key limitations of existing aging systems by providing a human-specific, physiologically relevant, and experimentally tractable platform. Unlike many current models, it is amenable to both genetic manipulation and chemical screening, enabling systematic interrogation of aging-related pathways. Furthermore, the model supports integrative multi-omics analyses within a controlled experimental context, allowing for in-depth exploration of the dynamics between epigenetic regulation, transposable element regulation, and inflammatory signaling during aging.

Despite its strengths, the hTSC–STB platform has several limitations that warrant careful interpretation. Firstly, it models differentiation-associated senescence in a specialized trophoblast lineage, and placenta-specific factors may not fully represent aging mechanisms in postmitotic or nonplacental proliferative cells. Secondly, the platform captures a programmed, adaptive senescence process that supports placental function, distinguishing it from the stochastic, decades-long accumulation of damage characteristic of organismal aging. While the system provides unique insights into epigenetic and retrotransposon-driven senescence, its findings should be validated in complementary human aging models to avoid overgeneralization of developmental pathways as universal aging drivers.

Collectively, our findings redefine trophoblast development as a programmed, “aging-like” process rather than a stochastic or purely degenerative event [1]. Although the placenta-specific nature may limit the direct extrapolation of these findings to other tissues, the presence of conserved aging hallmarks in this system underscores its value as a robust and physiologically relevant platform. It provides a unique opportunity to investigate fundamental mechanisms of cellular aging and to assess potential therapeutic interventions in a human-relevant context (Fig. 1).

Future studies should aim to validate candidate molecular regulators identified in the hTSC-STB primary screens using complementary models of aging and evaluate the translational potential of this system in the context of aging-related diseases. By bridging the fields of stem cell biology and developmental biology to gerontology, and by integrating epigenomic insights from studies on stem cell aging, this model offers a unique platform to accelerate the discovery of key aging regulators and the development of novel therapeutic strategies. In doing so, it contributes to the broader goal of promoting healthy longevity and addressing the molecular underpinnings of aging across tissues.
